# East Meets West: Addressing Frailty and Sarcopenia

**DOI:** 10.1093/geroni/igaf122.1278

**Published:** 2025-12-31

**Authors:** Sean Leng, Cuntai Zhang, Barbara Resnick

## Abstract

Frailty and sarcopenia are two critically important geriatric syndromes. As China and the US each face large and fast-growing old adult populations, substantial efforts have been devoted to study frailty and sarcopenia in both countries. This East Meets West symposium will present most recent and exciting research progress by senior investigators from China and in the US. Dr. Lin Kang will present results from a randomized trial demonstrating the feasibility and beneficial impact of a 6-month intervention of WHO ICOPE care pathways on health outcomes measures including vitality and mobility in frail and pre-frail older adults. Dr. Kathryn Callahan will describe an electronic frailty index (eFI) as a passive digital marker for frailty. Dr. Sean Leng will present the work from his group on bidirectional relationship between frailty and viral infections in older adults. Dr. Yaomin Hu will present her effort using self-developed AI diagnostic system and other technological innovations to establish a database and biobank for sarcopenia in China. Dr. Zhen Liang will present data suggesting that KLF13 restrains Dll4-muscular Notch2 axis to improve sarcopenia in animal models. Dr. Roger Fielding will describe the ongoing efforts towards a global definition and the potential for therapeutic interventions for sarcopenia in older adults. Dr. Cuntai Zhang, President of Chinese Medical Association Geriatrics Branch (CMA-GB) will serve the Co-Chair of this symposium. Dr. Barbara Resnick, President Emeritus of the Gerontological Society of America (GSA) will serve as the discussant.

